# Switchable Kirigami Structures as Window Envelopes for Energy-Efficient Buildings

**DOI:** 10.34133/research.0103

**Published:** 2023-04-17

**Authors:** Hanzhi Yin, Xishu Zhou, Zhengui Zhou, Rong Liu, Xiwei Mo, Zewen Chen, Erqi Yang, Zhen Huang, Hao Li, Hao Wu, Jun Zhou, Yi Long, Bin Hu

**Affiliations:** ^1^ Wuhan National Laboratory for Optoelectronics, School of Optical and Electronic Information, Huazhong University of Science and Technology, Wuhan, Hubei 430074, P. R. China.; ^2^ School of Mechanical Science and Engineering, Huazhong University of Science and Technology, Wuhan, Hubei 430074, P. R. China.; ^3^ Department of Electronic Engineering, The Chinese University of Hong Kong, Shatin, New Territories, Hong Kong SAR 999077, P. R. China.; ^4^ School of Materials Science and Engineering, Nanyang Technological University, Singapore 639798, Singapore.; ^5^ Shenzhen Huazhong University of Science and Technology Research Institute, Shenzhen 518057, P. R. China.

## Abstract

Efficient regulation of thermal radiation is an effective way to conserve energy consumption of buildings. Because windows are the least energy-efficient part of buildings, their thermal radiation regulation is highly demanded, especially in the changing environment, but is still a challenge. Here, by employing a kirigami structure, we design a variable-angle thermal reflector as a transparent envelope of windows for their thermal radiation modulation. The envelope can be easily switched between heating and cooling modes by loading different pre-stresses, which endow the envelope windows with the ability of temperature regulation, and the interior temperature of a building model can be reduced by ~3.3 °C under cooling mode and increased by ~3.9 °C under heating mode in the outdoor test. The improved thermal management of windows by the adaptive envelope provides an extra heating, ventilation, and air-conditioning energy savings percentage of 13% to 29% per year for buildings located in different climate zones around the world, making the kirigami envelope windows a promising way for energy-saving utilization.

## Introduction

Nowadays, building energy consumption as one of the largest energy consumption terminals in the whole world occupies about 32%, and even 40% in developed countries (i.e., 39% in the United States and 40% in Europe [1–3]). Among these, 50% of building energy consumption is applied to building heating, ventilation, and air-conditioning (HVAC) systems [4], which directly reveals the huge demand for building space temperature control. Developing innovative technologies to save energy consumption is essential [5]. Compared with roofs, floors, and walls, heat loss associated with windows accounts for up to 40%, making it the least energy-efficient element of buildings [6]. Therefore, exploring a simple and efficient strategy to optimize the thermal performance of windows is essential for energy-efficient buildings.

Isolating thermal conduction and convection is a useful solution for window thermal management such as using multi-paned windows [7], and some emerging materials such as nanoporous silica aerogels and transparent wood [8,9] have also been proposed as potential alternatives to glazing. Another vital approach is to adjust the radiative heat exchange of windows with its surroundings, especially with the cold universe because of the high emissivity of the long-wave infrared (ε_LWIR_) of the window [10]. However, current methods for window radiation control are mostly static and lack adaptability to the ever-changing weather conditions [11,12], such as sputtering low-emissivity (Low-E) coating [11]. The fixed feature is suitable for the buildings located in the regions without marked temperature fluctuations; however, for the hot summer and cold winter (HSCW) zones and even hot daytime and cold nighttime regions, the high ε_LWIR_ window favored in hot time for cooling energy saving may lead to extra heating energy consumption in the cold time [12]. Thus, exploring a strategy of dynamic radiative modulation of windows is helpful to energy-efficient buildings.

Motivated by this demand, thermochromic and mechanochromic phase-change materials have been developed as window coating for ε_LWIR_ modulation [13–16]. Nevertheless, because the thermal emission of the planar windows is omnidirectional, the radiative heat exchange efficiency of the windows, such as huge glazing curtain walls, is angular dependent. Therefore, manipulation of radiation angle of windows is important to alleviate or enhance the thermal radiation blocking effect for cooling or heating, especially for short buildings in urban densely tall building clusters [17]. Moreover, most regions receive solar radiation at various angles in different seasons; adaptively solar modulation is also required for lighting and thermal management [18].

Compared to redesigning the structure of the window or adopting sophisticated techniques such as gradient epsilon-near-zero materials [19] or thermal mirror hole devices [17,20–22], it is more feasible to add a window envelope to regulate the radiation angle, which has been demonstrated to actively modulate solar radiation through a machine control system [23]. In contrast, the kirigami-based structures can be a simpler way to achieve this function because of the excellent shape-programmable and reconfigurable characters [24–29] and they have been exploited for dynamic light manipulation recently [25,27,29–31].

Here, we introduce a kirigami pattern into a transparent thermal reflecting film and design a window envelope for effective dynamic radiation modulation. The kirigami envelope can adjust the angles of omnidirectional thermal emission of the planar windows by stretching and can switch between cooling and heating modes by loading different pre-stresses, showing the potential adaptive energy-saving capability to the changing weather conditions. The outdoor experiments show that the kirigami envelope window can effectively regulate the interior temperature of a building model, which can achieve ~3.3 °C reductions under cooling mode compared to that using a bare window and can increase ~3.9 °C above ambient temperature under the heating mode, and the flexible thermal modulation ability of the kirigami envelope window could bring an extra HVAC energy-saving percentage of 13% to 29% per year for buildings located in different climate zones according to the simulation.

## Results and Discussion

### Principle of kirigami-based thermal radiation modulation

**Fig. 1.**
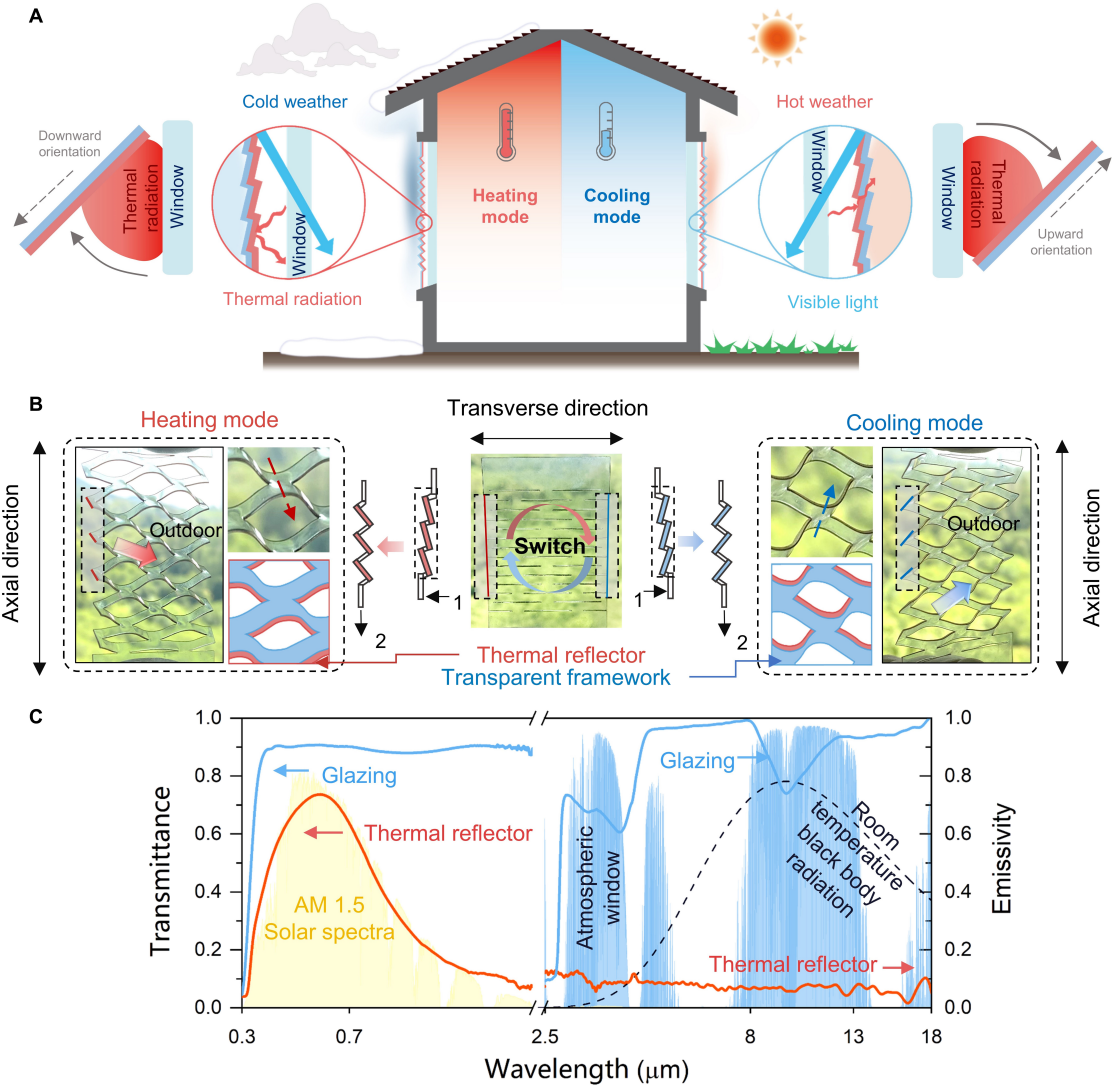
Concept and design principle of the dual-mode kirigami envelope. (A) Schematic illustration of different application scenarios and single unit of the kirigami envelope. (B) Mode switch of the envelope by pre-stress and axially stretching for different bridge orientations. (C) Transmittances of the thermal reflector and glazing over ultraviolet-visible-near infrared region and corresponding ε_LWIR_, and AM 1.5 solar spectrum (yellow region), a typical atmospheric window (blue region), and the heat radiative band of the black body of ∼300 K (dashed black curve).

The strategy of thermal radiation modulation of window using a transparent kirigami envelope is illustrated in Fig. 1. The thermal reflectors (red layer; i.e., Low-E) coated on the flexible transparent framework (blue layer) face the windows (Fig. 1A), which can manipulate the angles of omnidirectional thermal radiation emitted from the windows by adjusting the orientation of the bridges in kirigami envelopes, endowing the kirigami envelopes with the ability of mode switch between heating and cooling. The enlarged single-unit schematic of the dual-mode kirigami structure is shown in Fig. 1A. Specifically, under the heating mode (left schematics in Fig. 1A), the bridges of the kirigami structure deflect downward (Fig. 1B, red dashed arrow), ensuring that the thermal reflector faces down to block the radiative heat loss of the window under a cold weather condition. In contrast, the bridge structure deflects upward and the thermal reflector faces up for cooling demand (Fig. 1B, blue dashed arrow) to maximize the heat dispersion of the window under a hot weather condition. Stretching the kirigami envelope along the axial direction leads to mechanical instability and initiates a controlled bucking process in the transverse direction. As shown in Fig. 1B, the mode of the kirigami envelopes can be controlled by applying different pre-stresses; lifting or lowering one end of the envelope (step 1) would cause a slight tilt in different directions. The following stretching (step 2) induces out-of-plane deformations of the entire kirigami envelopes, and the deflect angles can be tuned using a loaded tensile strain.

The materials of the kirigami envelope were selected by considering emissivity and mechanical robustness. A visible–transparent tin-doped indium oxide (ITO)-coated polyethylene terephthalate (PET) film was used as a thermal reflector, and an elastic polydimethylsiloxane (PDMS) layer was used as a framework to enhance the mechanical reliability during the repeated stretching of the kirigami structure [32–36]. Different kirigami patterns were designed and fabricated on the ITO/PET/PDMS films by laser processing (Methods). Figure 1C shows the transmittance and ε_LWIR_ of the fabricated kirigami envelope and the glazing, in which the glazing as the emitter has a high ε_LWIR_ of 0.91, while the thermal reflector ITO exhibits an extremely low ε_LWIR_ of 0.07 (Fig. S1), both of them are highly transparent in the visible region to ensure the daytime indoor light (Fig. S2), and the laser cutting process has a negligible effect to the optical properties (Fig. S3).

### The design of the kirigami envelope for optimal radiation angle

**Fig. 2.**
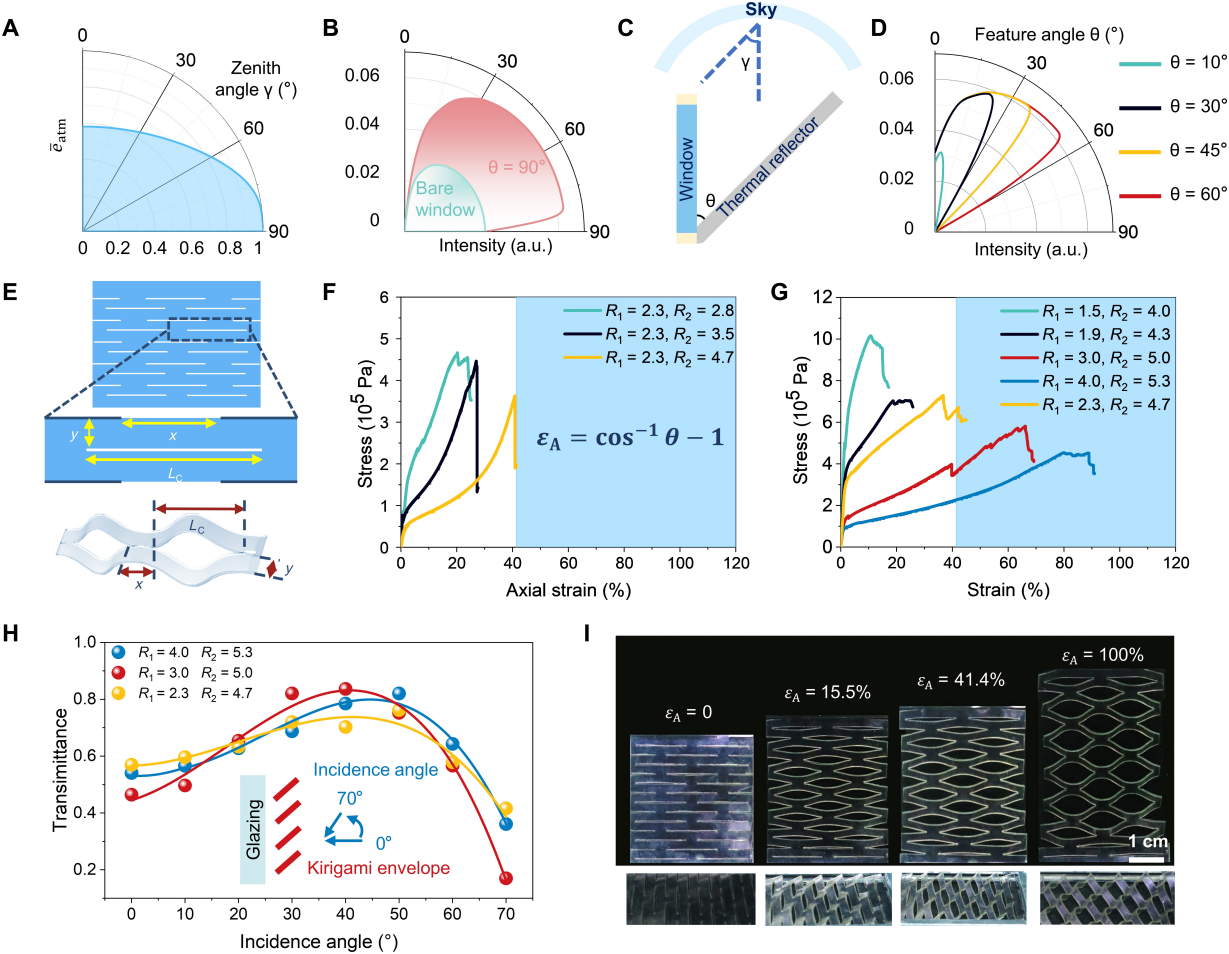
The design of optimal kirigami structure with radiation angle modulation. (A) The simulated emissivity of atmospheric radiation received by a vertically placed window at different zenith angles. (B) The simulated angular radiative intensity distribution of a bare window (green curve) and the window with a horizontal thermal reflector (pink curve). (C) Vertically placed window and thermal reflector as simplified geometry diagram of the kirigami structure. (D) The thermal radiation intensity of the window with thermal reflector placed at different feature angles. (E) Key structural parameters of the kirigami structure. (F and G) Stress–strain curves of kirigami structures with different *R*_1_ and *R*_2_. (H) *T*_lum_ of different kirigami structures along different incidence angles with the schematic illustration of the measurement in the inset. (I) The pictures of the stretched kirigami structure under different *ε*_A_.

To identify the influential factors on the radiative intensity of windows, the average emissivity ($\bar{e}$_atm_) of atmospheric radiation received by a vertically placed window at different zenith angles (γ) is given in Fig. 2A, which increases from 0.6 to 1 when γ increases from 0° to 90°. The result indicates that the atmospheric transmittance decreases with the increase of γ, especially when γ is greater than 45°; the transmittance decays rapidly (Fig. S4); and the atmospheric window completely closed at γ of 90°. The relationship between γ and atmospheric transmittance preliminary elucidates that the modulation of the radiation direction can enhance the passive cooling efficiency of windows by launching the thermal radiation into space along the direction with high atmospheric transmittance, and vice versa for the windows that need inhibiting heat loss.

On the other side, the thermal radiation intensity of vertically placed windows is simulated, and the omnidirectional hemispherical distribution of a bare window is shown in Fig. S5. By introducing a horizontal thermal reflector (θ = 90^o^) into the bare window (Fig. S6), the radiation intensity of the window is no longer omnidirectional, and the angle-dependent distribution is plotted in Fig. 2B, which indicates that the thermal radiation can be well-regulated and concentrated in a small region toward the sky compared to that of the bare window [20]. On the basis of this modulation rule, a simplified kirigami structural model is fabricated (Fig. S7), with tunable feature angles (θ) from 0° to 90° as schematically shown in Fig. 2C. The range of radiation modulation of the model is plotted in Fig. 2D, and the results show that the radiation intensity increases sharply when θ increases from 0° to 45° but gradually becomes saturated in all directions with a further increase from 45° to 60°. Considering that the atmospheric transmittance decays rapidly when γ surpasses 45° as shown in Fig. 2A, the θ of 45° is the optimal angle that can achieve maximum thermal radiation intensity through the atmosphere.

To obtain the kirigami structure that can satisfy the designed capability in radiation modulation and simultaneously maintain mechanical stability during repeated deformation, a simple but effective kirigami pattern for the window envelope is shown in Fig. 2E. The geometrical parameters consist of the transverse cut length *L*_C_, the transverse cut spacing *x*, and the axial layer spacing *y*. The patterned structure was subjected to 2D–3D deformation when stretched along the axial direction and contracted in the transverse direction; the equivalent geometric model of the kirigami structure is shown in Fig. S8. The expression of θ is defined in Fig. 2F inset, where *ε*_A_ denotes the strain along the axial direction after stretching, and 2 dimensionless parameters *R*_1_ and *R*_2_ are defined as *L*_C_/*x* and *L*_C_/*y*, respectively, to simplify the expressions. The specific calculation procedure is shown in Note S1 according to the study of Lamoureux et al. [29].

Different samples with varied *x*, *y*, and *L*_C_ in Tables S1 and S2 were fabricated and compared to determine the optimal structural parameters of the kirigami unit. We performed stress tensile tests on the prepared samples to screen the ones that achieve the optimal feature angle θ. According to the inserted equation in Fig. 2F that establishes the relationship between *ε*_A_ and θ, the minimum ultimate axial strain *ε*_A_ needs to exceed 41.4% to ensure that θ can reach 45°. For the fixed *x* and *L*_C_ (i.e., fixed *R*_1_ = 2.3), only the yellow curve (*R*_2_ = 4.7) can approach the marked blue area, which represents the desired axial strain ranges. More elaborate experiments were investigated by varying *R*_2_ near 4.7 to refine the structural model, and additional red (*R*_1_ = 3, *R*_2_ = 5) and blue (*R*_1_ = 4, *R*_2_ = 5.3) curves also meet the *ε*_A_ requirement as shown in Fig. 2G. In addition, the relationship between θ and *ε*_A_ with variable structural parameters is also established in Fig. S9, which confirms that θ only depends on *ε*_A_ and is independent of *x*, *L*_C_, and *y* (Note S1) [29].

The intrinsic high visible transmittance of the materials makes the kirigami envelope highly transparent, and the gradually formed openings during stretching can provide additional incident channels of the sunlight for sufficient indoor lighting to meet the energy-saving requirements of the buildings. The visible transmittances of 3 qualified samples in Fig. 2G were measured with optimal θ of 45^o^ under a fixed *ε*_A_ strain. The incident angle of the light increased gradually as shown in the inset Fig. 2H, and the visible transmittances of all the samples increase with incidence angles but achieve the maximum values at different angles. Among them, the blue line sample exhibits the optimal incidence angles of 50° exceeding the optimal θ of 45°, which simultaneously ensures the highest visible light transmittance and best radiation intensity along the zenith direction. Therefore, on the basis of the evaluation of the above comprehensive performance, the blue line sample with structural parameters *L*_C_, *x*, and *y* of 16, 4, and 3 cm, respectively, is selected as the optimal window envelope in the following experiments. Figure 2I demonstrates the corresponding pictures of the fabricated kirigami structure, which is stretched under different *ε*_A_ to demonstrate the feasibility of dynamic modulation.

### Heating and cooling effect of the kirigami-based structure

**Fig. 3.**
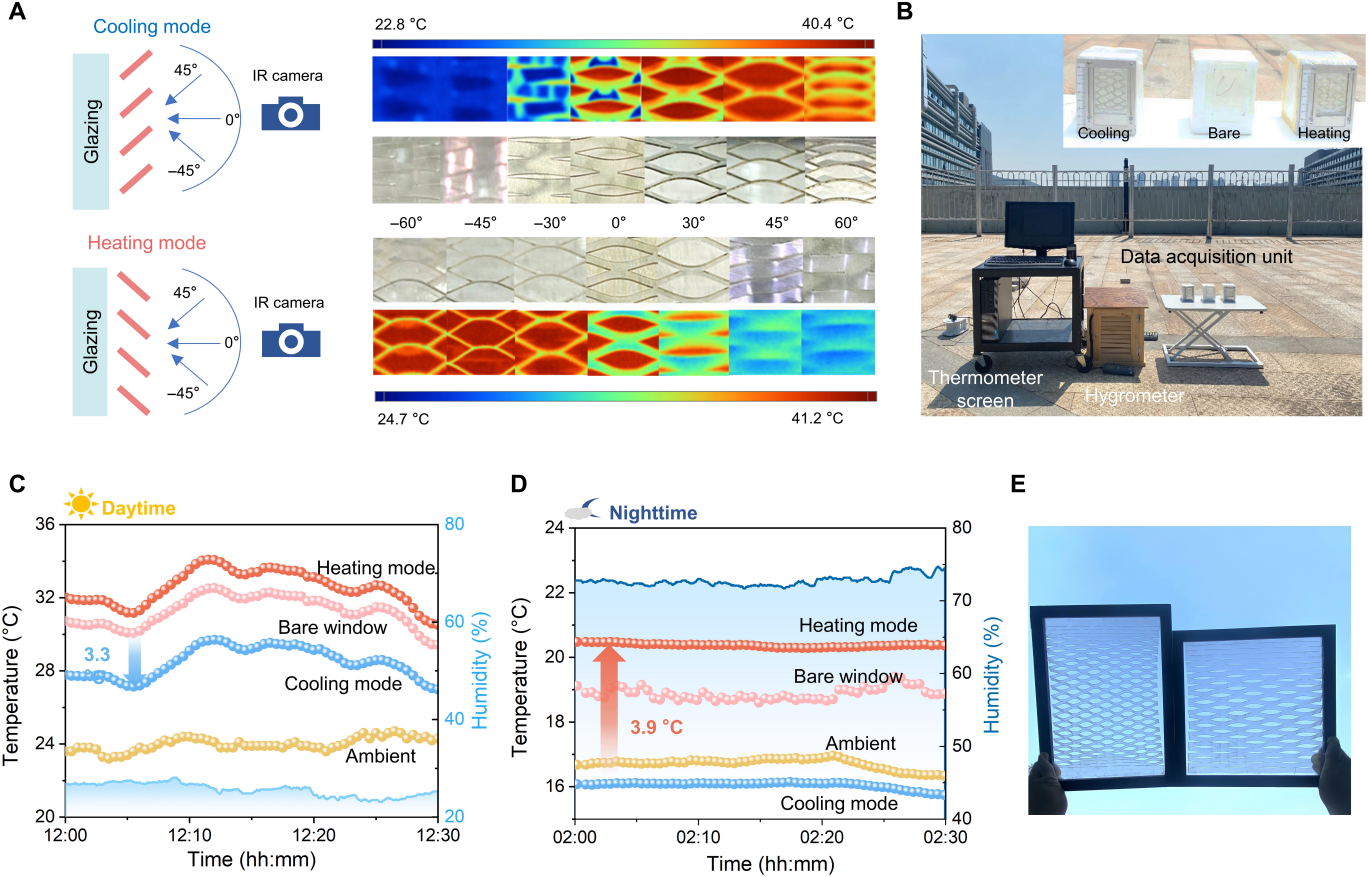
The practical performance of the kirigami envelope windows. (A) Schematic of the temperatures examined along different observation angles. Optical photographs and thermal images of the kirigami envelope under heating and cooling modes. (B) Photo of the experimental apparatus on an outdoor platform. (C) Outdoor performance of the kirigami envelope under cooling mode during daytime in Wuhan, China on 2022 October 19. The solar irradiance was about 720 to 750 W/m^2^. (D) Outdoor performance of the kirigami envelope under heating mode during nighttime in Wuhan, China on 2022 March 11. (E) Photograph of large kirigami envelope windows with a size of 20 cm × 30 cm and 25 × 25 cm. IR, infrared.

The effect of the kirigami envelope on the thermal radiation of windows was directly visualized using an infrared camera as shown in Fig. 3A. The kirigami envelope was stretched to the heating or cooling mode and installed on a heated glazing (40 °C) as schematically shown in Fig. 3A, and the apparent temperatures of the stretched envelope from different observation angles were recorded simultaneously. In the heating mode, the opening area of the envelope gradually disappeared in the field of view when the observation angle increased along the counterclockwise direction, while the blue infrared images indicate that the thermal radiation of the window can be effectively blocked along the observation direction. In contrast, in the cooling mode, more openings are exposed and the infrared images become redder, indicating that the thermal radiation emit without obstacles along the observation direction.

We further performed an outdoor experiment on an open-air platform in Wuhan. Figure 3B shows the photo of the experimental setup. Three foam chambers with vertically installed windows were used as building models and placed closely, and the insulated box minimizes the parasitic heat exchange of the system with surroudings. Two kirigami envelopes set as heating and cooling modes were covered on 2 windows, and the middle one kept the bare window as the control model. The thermocouples were placed inside the chambers to monitor the temperatures, and the hydrometer was placed on the thermometer screen to measure the temperature and humidity of the surroundings. The temperature variations of the ambient (yellow line), bare-window model (pink line), cooling-mode envelope model (blue line), and heating-mode envelope model (red line) during daytime (Fig. 3C) and nighttime (Fig. 3D) within a day were recorded. The daytime temperatures of all the models were higher than ambient temperature because of the inevitable solar light absorption of the interior chamber when the solar irradiance was over 720 W/m^2^, but the cooling-mode envelope model can maintain a notable temperature drop of ~3.3 °C (blue line in Fig. 3C) compared to the bare-window model, confirming the notable passive cooling effect due to the enhanced thermal radiation intensity of the sky, which can even cool down the model below ambient temperature at nighttime (blue line in Fig. 3D). While the heating-mode envelope can better conserve the heat and the model achieve a higher temperature (red line in Fig. 3C) compared to the bare one at daytime, and heat dispersion can also be eliminated during nighttime (red line in Fig. 3D) and the model retains ~3.9 °C above ambient temperature, experimentally proving that radiation heat loss can be inhibited effectively. Figure 3E shows photography of a window containing the kirigami envelope with the size of 20 cm × 30 cm and 25 × 25 cm, demonstrating practical potential in large-scale applications (Methods).

We also demonstrated the good water resistance of the envelope material in the outdoor test (Fig. S10).

### Energy saving simulation

**Fig. 4.**
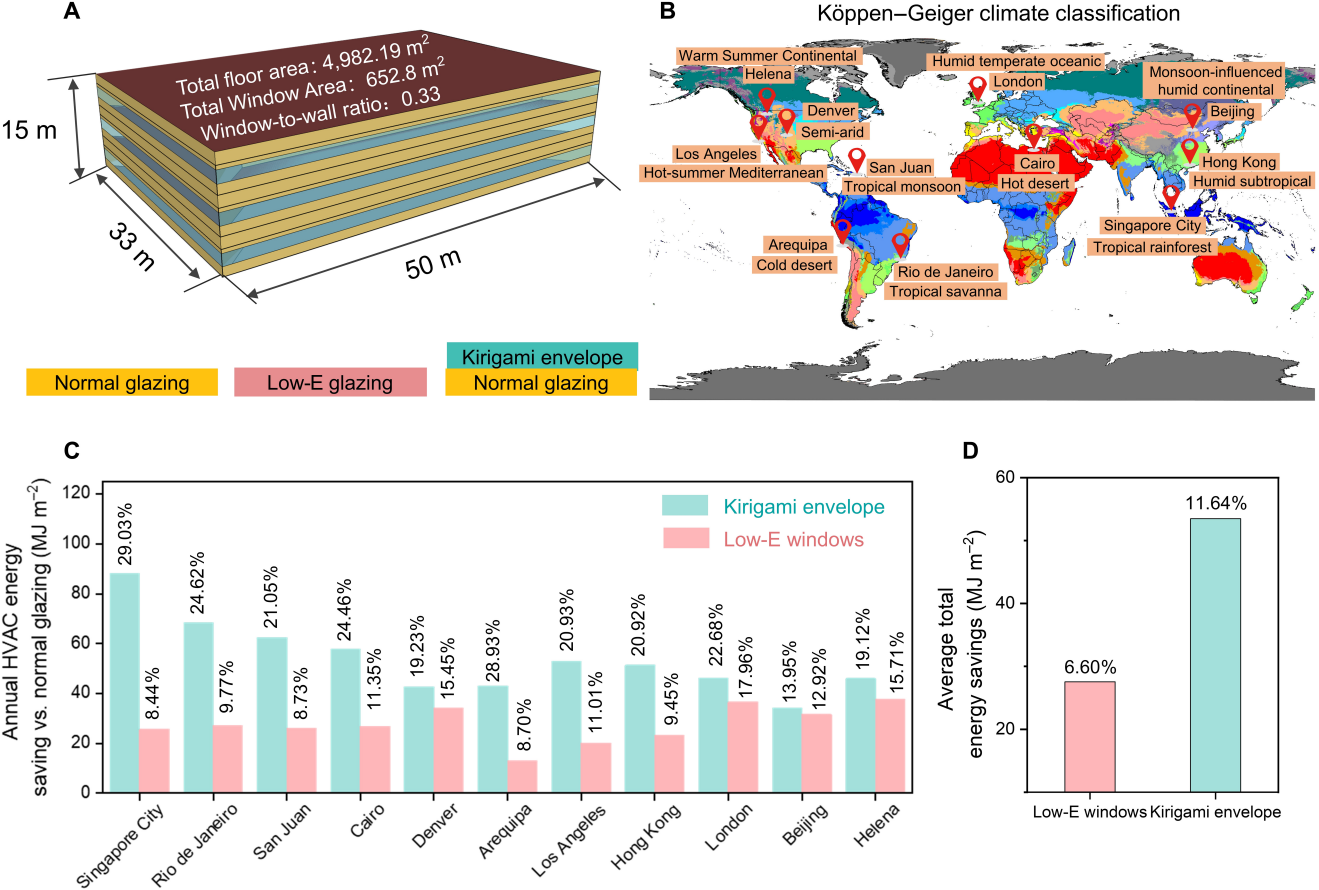
HVAC energy-saving assessment around the world. (A) Schematic representation of the medium office building. Three kinds of windows are used to simulate the dynamic energy-saving effect. (B) Representative cities with different climatic conditions based on the Köppen–Geiger climate classification for energy saving simulation. (C) Annual HVAC energy saving and (D) average total energy savings of the buildings with the kirigami envelope windows and Low-E windows compared to that using normal windows in representative cities of different climate zones.

On the basis of the measured performance of the dual-mode kirigami envelope, the energy-saving potentials in building applications were evaluated using EnergyPlus. The representative building model for simulation is a medium office with a floor area of 4,982 m^2^ and a window-to-wall ratio of 33% as shown in Fig. 4A. We simplified the kirigami structure and propose the equivalent model as Venetian blinds (Fig. S11), and the corresponding optical and thermal parameters used in the simulation are listed in Table S3. We also calculate the conductivity of the composite material considering the effect of non-radiative heat transfer, and thermal openness is used to quantify convective heat transfer through the equivalent shading system (Methods).[37]

To investigate the effect of kirigami envelopes in building energy saving, 3 kinds of windows were fabricated, including normal glazing (CLEAR 6MM), commercial Low-E glazing (LoE CLEAR 6MM), and normal glazing with kirigami envelopes as illustrated in Fig. 4A. We selected 11 representative cities located in different climate zones, covering 4 main climate classification classes and 11 subtypes based on the Köppen–Geiger climate classification for comparison as shown in Fig. 4B [38], and the corresponding specific weather conditions and locations are listed in Table S4. External boundary conditions are based on the local weather conditions (i.e., building surface temperature, wind speed, and sunlight), and the internal air temperature is based on the medium office hourly operation schedules of the U.S. Department of Energy commercial reference building models of the national building stock [39].

The extra annual HVAC energy savings in 11 cities due to the utilization of dual-mode kirigami envelopes are shown in Fig. 4C ranging from 34 to 88 MJ/m^2^, including Singapore City, Singapore (88.13 MJ/m^2^); Rio de Janeiro, Brazil (68.49 MJ/m^2^); San Juan, Puerto Rico (62.53 MJ/m^2^); Cairo, Egypt (57.72 MJ/m^2^); Denver, USA (42.62 MJ/m^2^); Arequipa, Peru (43.12 MJ/m^2^); Los Angeles, USA (52.81 MJ/m^2^); Hong Kong, China (51.33 MJ/m^2^); London, UK (46.17 MJ/m^2^); Beijing, China (34.14 MJ/m^2^); and Helena, USA (46.01 MJ/m^2^), which could account for up to 13% to 29% per year compared with normal windows for a building located in different climate zones around the world. The energy-saving results exceed the performance based on commercial Low-E windows, which provide a relatively inferior percentage of 8% to 15% per year compared with normal windows due to the limitation of static thermal radiation. It is worth mentioning that, for the building located in different climate-representative cities, the monthly HVAC energy consumptions show that the Low-E windows demonstrate good thermal insulation in cold months (Fig. S12A). The envelope can also be closed entirely as Low-E windows at nighttime or the heating demand is greater than the light demand in cold areas. However, the Low-E window possesses a higher consumption when the building demands to cool (Fig. S12B), proving the substantial energy-saving contribution of the dynamic radiation modulation. Therefore, the dual-mode kirigami envelope exhibits superior energy-saving performance in different seasons. The lighting energy consumption of 3 kinds of windows including normal glazing, kirigami envelopes, and commercial Low-E glazing are also compared in Fig. S13, which shows that the increase in lighting energy consumption due to the introduction of the kirigami envelopes can be negligible. The average total annual energy savings of Low-E windows and kirigami envelope building models across the above 11 cities are 27.55 MJ/m^2^ (6.60 %) and 53.47 MJ/m^2^ (11.64 %), respectively. In actual application, the modulation frequency of the envelopes can be increased according to the ever-changing weather or zenith angles to further improve the energy-saving performance [14].

## Methods

### Materials and fabrication

The commercial ITO-deposited PET films were purchased from South China, Xiangcheng Technology Co. Ltd. The PDMS is purchased from Dow Silicones Corporation. As shown in Fig. S14, the ITO-PET film was fixed on the substrate, and PDMS with the pre-polymer and cross-linker in a ratio of 10:1 was spin-coated on the PET side at 250 rpm/s for 10 s (first stage) and at 500 rpm/s for 50 s (second stage) on a homogenizing spin-coater (Jiangsu Leibo). Then, the coated film was placed in an oven (Suzhou Jiangdong DHG-9145A) and cured at 60 °C for 3 h. The kirigami envelopes were fabricated using a laser engraving and cutting machine (Guangzhou Huazun ILS-3V), and the laser power parameter was set to 30% and the speed parameter to 3 mm/s (Fig. S15). The big-scale sample was fabricated using the blade-coating PDMS layer on ITO-PET composite film.

### Characterization

The visible transmittance and near-infrared reflectance of the samples were measured using a ultraviolet-visible spectrophotometer (Shimadzu, UV-3600 Plus, Japan), and the mid-infrared emittance was measured using a Fourier transform infrared spectrometer (Thermo Fisher Scientific, Nicolet-is50, USA) with an infrared integrating sphere attachment (PIKE).

The average visible transmittance and ε_LWIR_ of the experimental results can be calculated by the following equations, respectively.

$$T_{\mathrm{lum}}=\frac{\int_{0.3}^{0.78}d\lambda \cdot T\left(\lambda \right)\cdot I_{\mathrm{AM}1.5}\left(\lambda \right)}{\int_{0.3}^{0.78}d\lambda \cdot I_{\mathrm{AM}1.5}\left(\lambda \right)}$$ (1)

$$\varepsilon_{\mathrm{LWIR}}=\frac{\int_{2.5}^{18}d\lambda \cdot \varepsilon \left(\lambda \right)\cdot I_{\mathrm{BB}}\left(T,\lambda \right)}{\int_{2.5}^{18}d\lambda \cdot I_{\mathrm{BB}}\left(T,\lambda \right)}$$ (2)

where *T*(λ) denotes visible transmittance in the wavelength range of 300 to 780 nm, *ε*(λ) notes the emissivity in the wavelength range of 2,500 to 18,000 nm, *I*_AM1.5_(λ) denotes the energy density of solar radiation at a wavelength, and *I*_BB_ (*T*, λ) denotes the energy density of black body radiation. Solar transmittances of the original film and corresponding kirigami envelope are shown in Fig. S3. The *T*_lum_ of the composite material is 0.72, and the *T*_lum_ of the kirigami envelope obtained after laser etching is 0.66, which meets the requirement of the window for indoor lighting. The *T*_lum_ decreases by 0.06 after laser treatment because of the increased surface roughness during the laser cutting process, which enhanced the light scattering. The tensile stress–strain test of the kirigami structure used a microcomputer-controlled electronic universal testing machine (Rigel, Shenzhen).

### Indoor and outdoor test

The infrared images were taken by the infrared camera (Fluke, TiX520, USA), and the hot stage (IKA, C-MAG HP7, German) was set at 40 °C. The foam boxes were used as building models in outdoor experiments, and the normal glazing (6MM) was vertically fixed on the boxes. The foam boxes and corresponding chamber sizes were 9.5 × 7 × 7 cm^3^ (height × width × depth) and 4 × 5.5 × 5.5 cm^3^ (height × width × depth), respectively. The window is 5.5 × 7 × 0.6 cm^3^ (length × width × thickness). Two models were installed with kirigami envelopes. Two kirigami envelopes were stretched with tensile strains of 41.4% and set as different modes of cooling mode and heating mode, respectively. The middle control model was installed with a bare window. K-type thermocouples (OMEGA TT-K-36-SLE) were placed inside the model chambers and the thermometer screen for monitoring the temperature of the samples and surroundings, respectively. The thermocouples were connected to a computer via a data collector (PICO TC-08) for real-time temperature recording. A hydrometer (Center 310 Rs-232) was used to measure and record the temperature and humidity of the environment. The probes were fixed inside the thermometer screen to reduce unnecessary interference. In addition, to mitigate the environmental effects on the heat convection and heat conduction of the experimental setup, a single layer of polyethylene film was used to encapsulate the constructed building model.

### Energy saving simulation

The window and building models were coupled and imported into EnergyPlus (version 9.1.0) for simulation calculation. The inner surface convection algorithm used the TARP method, and the outer surface convection algorithm used the default DOE-2 method. In addition, the heat transfer equation was used as the heat balance equation, and the simulation time step was set to 10 min. The basic parameters of the normal glazing (CLEAR 6MM) and Low-E glazing (LoE CLEAR 6MM) were obtained from the database of EnergyPlus. The monthly HVAC energy consumption was simulated based on the entirely closed kirigami envelope windows in cold months in Denver. The thermal conductivity had almost no impact on energy performance because the envelopes are not in physical contact with the window. Thermal openness was also called the permeability factor; it is the airflow permeability for the material in the shading system and is used to quantify convective heat transfer through the shading layer [37], i.e., the fraction of the shade surface that is open to airflow.

In the daylighting and lighting simulation, the lighting control model has incorporated daylighting controls by implementing daylighting reference points in all thermal zones. The models were created by the U.S. Department of Energy, and we adopted the SplitFlux methods [39]. In addition, the average power density was set as 8.50 W m^−2^ based on the zone floor area according to the ASHRAE Standard and Guidelines [40]. The illuminance set point was 375 lux in the simulation model, exceeding the recommended minimum illuminance level of 300 lux for office space [41], and the stepped lighting control type was applied.

## Conclusion

In summary, by combining the kirigami technology and radiation modulation strategy, we propose the concept and design of a dual-mode window envelope. We found that the thermal radiation angles of the vertical windows can be effectively tuned by stretching the kirigami envelope to achieve heating and cooling modes. On the basis of the comprehensive evaluation of mechanical stretchability and angular-distributed thermal radiation intensity, the optimal parameters of the kirigami structure are determined to maximize the modulation capability and to maintain the highly visible transmittance for indoor lighting. Outdoor experiment results show that the building model with a cooling-mode envelope window can decrease ~3.3 °C compared to the bare window model at daytime, while the heating-mode envelope can keep ~3.9 °C above the ambient temperature at nighttime, which can provide an extra HVAC energy saving percent of 13% to 29% per year for a building located in different climate zones according to the energy consumption simulation. The results show that adding the window envelopes can be a feasible way to enhance or eliminate the heat dispersion dynamically for interior temperature regulation. The envelope can be updated by optimizing the kirigami materials, such as selecting smart framework materials that can respond to the changing weather, endowing the kirigami with practical opportunities for energy-efficient building.

## Data Availability

The data are available from the authors upon a reasonable request.

## Supplementary Materials

Supplementary Materials Figs. S1 to S15 Note S1 Tables S1 to S4
