# Supplementary material for: Switchable Kirigami Structures as Window Envelopes for Energy-Efficient Buildings
Source: Research (Wash D C). 2023 Apr 17;6:0103. doi: 10.34133/research.0103 (PMC10202178; doi:10.34133/research.0103)
Supplement: Supplementary Materials — Figs. S1 to S15 Note S1 Tables S1 to S4 [file research.0103.f1.docx]

Supplementary Materials

Switchable Kirigami Structures as Window Envelopes for Energy-Efficient Buildings

*Hanzhi Yin ^#1^, Xishu Zhou ^#1^,* *Zhengui Zhou ^1^, Rong Liu ^1^, Xiwei Mo ^1^, Zewen Chen ^1^, Erqi Yang ^1^, Zhen Huang ^1^, Hao Li ^2^, Hao Wu ^2^, Jun Zhou ^1^, Yi Long ^3,4^, Bin Hu^*1,5^*

*^1^* Wuhan National Laboratory for Optoelectronics, School of Optical and Electronic Information, Huazhong University of Science and Technology, Wuhan Hubei 430074, P. R. China

*^2^* School of Mechanical Science and Engineering, Huazhong University of Science and Technology, Wuhan, Hubei 430074, P. R. China

*^3^* Department of Electronic Engineering, The Chinese University of Hong Kong,

Shatin, New Territories, Hong Kong SAR 999077, P. R. China

*^4^* School of Materials Science and Engineering, Nanyang Technological University

Singapore 639798, Singapore

*^5^* Shenzhen Huazhong University of Science and Technology Research Institute

Shenzhen 518057, P. R. China


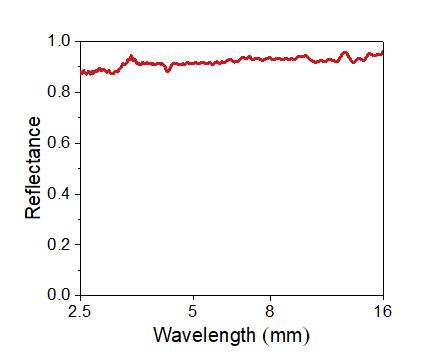


Fig. S1. The mid-infrared reflectance spectrum of ITO/PET film

Fig. S2. Transmittance spectra of the envelope window under different modes.


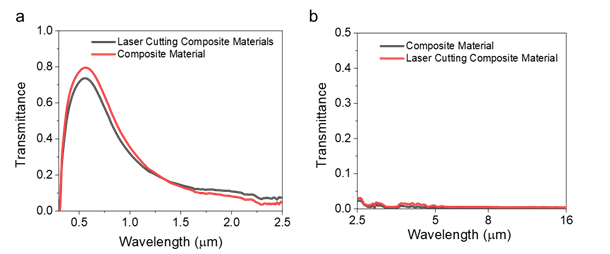


Fig. S3. a) Solar transmittance and b) Infrared transmittance spectra of the envelope material before and after laser cutting. The intrinsic visible transmittance is 0.72 and reduced to 0.66 after laser cutting because of the increased surface roughness.


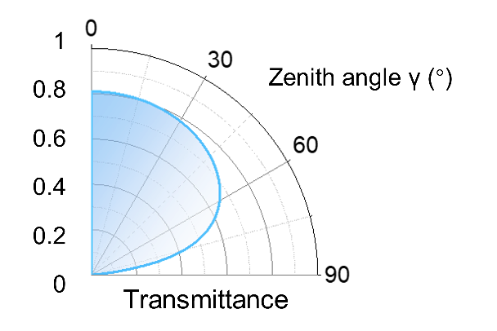


Fig. S4. Angular dependence of atmospheric transmittance.


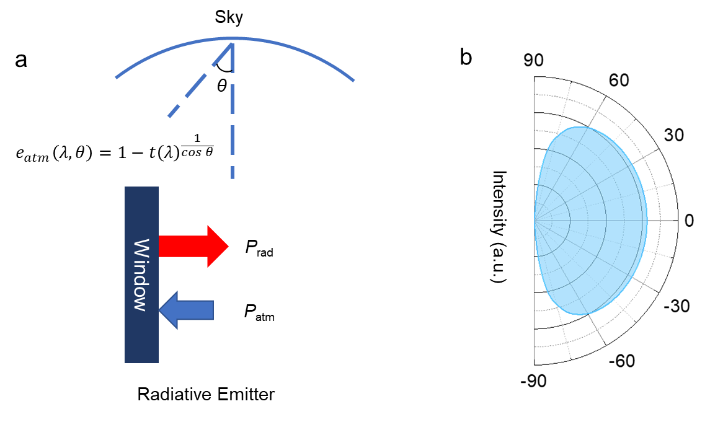


Fig. S5. The thermal radiation intensity of a vertically placed window and corresponding angular distribution of radiation intensity.


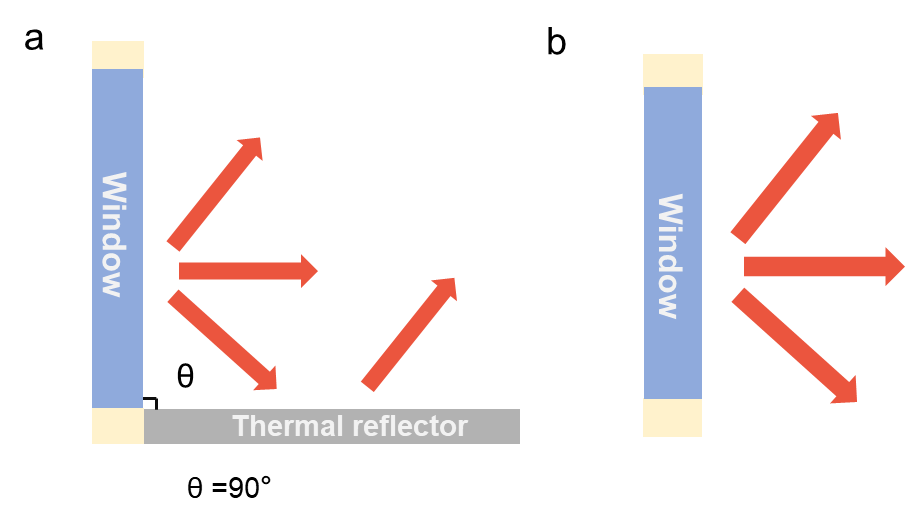


Fig. S6. Schematic of a bare window and the window with horizontal thermal reflector.


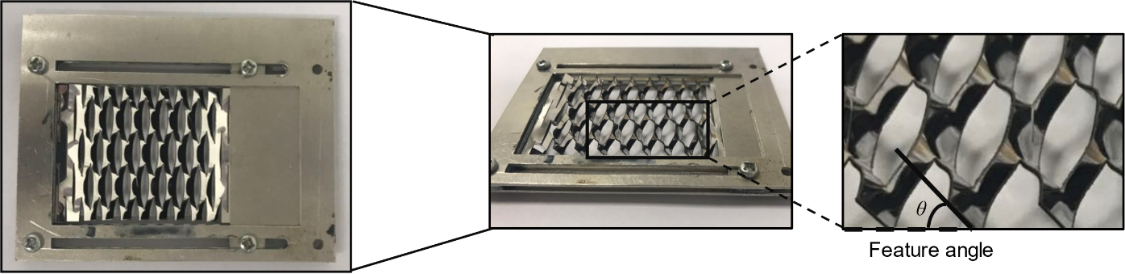


Fig. S7. Schematic of feature angle in kirigami structure.


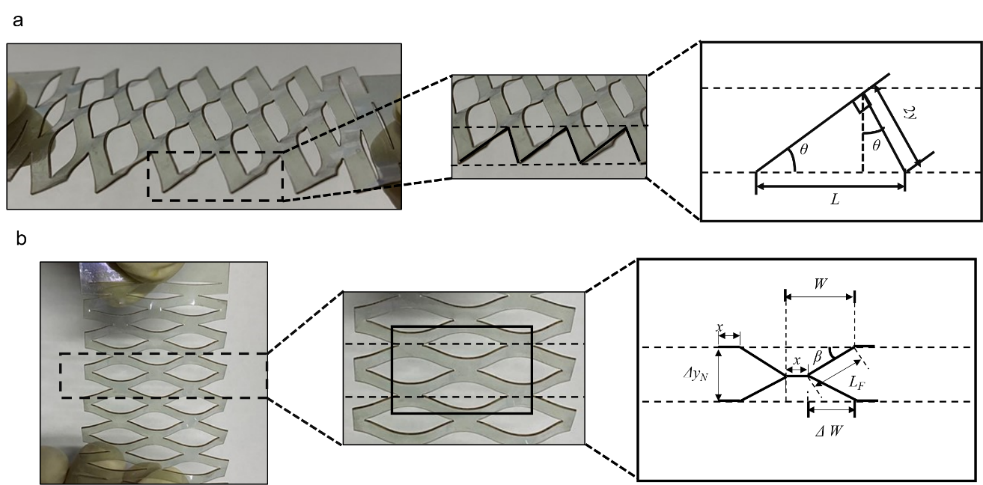


Fig. S8. Geometric diagram of axial and transverse tension of kirigami structure. (a) Derivation of geometric response along the axial direction. (b) Derivation of geometric response along the transverse direction. A detailed description of the parameters is listed in Note S1.

Note S1 Calculation of simplified geometric model of kirigami structure

𝜀_𝐴_ indicates the axial strain after stretching, which can be expressed by Equation S1.

| $\varepsilon_{A}$=$\frac{L-L_{0}}{L_{0}}$ | (Equation S1) |
| --- | --- |

As shown in Fig. S8a, 𝐿_0_ indicates the initial length of the kirigami structure in the axial direction when it is not stretched, and 𝐿 indicates the length along the axial direction after stretching. 𝜀_𝑇_ indicates the strain along the transverse direction after stretching, which can be expressed by Equation S2.

| $\varepsilon_{T}$=$\frac{W-W_{0}}{W_{0}}$ | (Equation S2) |
| --- | --- |

Similarly, *W*_0_ indicates the initial length of the kirigami structure’s transverse direction when it is not stretched, and *W* indicates the length along the transverse direction after stretching. From the geometric relationship in Fig. S8a, we know that,

| $L_{0}=2y$ | (Equation S3) |
| --- | --- |
| $L$=$\frac{2y}{\cos\theta}$ | (Equation S4) |

Bringing the equations S3 and S4 into equation S1, we obtain the expression of feature angle $\theta$

| $\theta=\cos^{-1} \left( \frac{1}{\varepsilon_{A}+1} \right)$ | (Equation S5) |
| --- | --- |

The change in the geometry of the kirigami structure in the transverse direction after stretching can be obtained from the geometric relations in Figure by the following equations.

| $W_{0}=x+L_{F}$ | (Equation S6) |
| --- | --- |
| $L_{F}=\frac{L_{C}-x}{2}$ | (Equation S7) |
| $W=x+\Delta W$ | (Equation S8) |
| $\Delta W=L_{F}\cos\beta$ | (Equation S9) |
| $\varepsilon_{T}=\frac{W-W_{0}}{W_{0}}=\frac{L_{F}\cos\beta-L_{F}}{x+L_{F}}$ | (Equation S10) |

To facilitate the representation of the calculation results two dimensionless parameters $R_{1}$= $\frac{L_{C}}{x}$and $R_{2}$= $\frac{L_{C}}{y}$are introduced and the simplified $\varepsilon_{T}$ expression is shown in equation S11.

| $\varepsilon_{T}=\frac{R_{1}-1}{R_{1}+1}\left\{ \cos\left[ \sin^{-1} \left( \frac{{2R}_{1}\tan\theta}{{R_{1}R}_{2}-R_{2}} \right) \right]-1 \right\}$ | (Equation S11) |
| --- | --- |

The limit of the maximum feature angle $\theta_{max}$ corresponding to the maximum axial strain of the kirigami structure depends only on the parameters $R_{1}$and $R_{2}$of the structural unit^[1]^.

| $\theta_{max}=\tan^{-1} \left( \frac{{R_{1}R}_{2}-R_{2}}{2R_{1}} \right)$ | (Equation S12) |
| --- | --- |

|  |  |
| --- | --- |


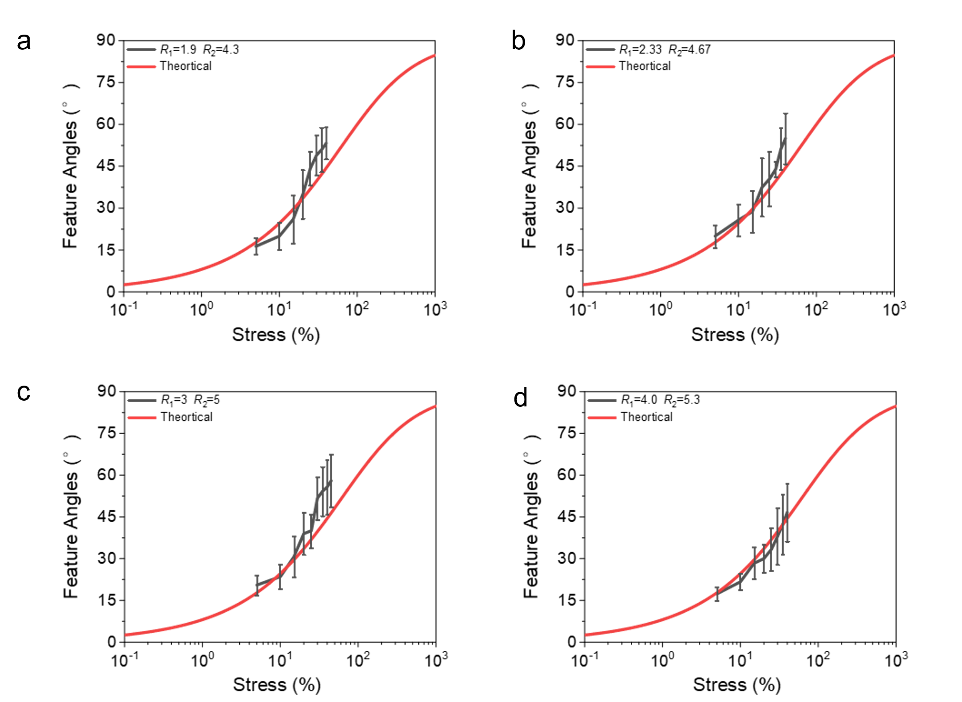


Fig. S9. Theoretical relationship and measured data between feature angle (θ) and axial strain (ε_A_). θ versus ε_A_ for different kirigami structures where *R*_1_ =1.9, *R*_2_ =4.3; *R*_1_ =2.3, *R*_2_ =4.7; *R*_1_ =3, *R*_2_ =5; *R*_1_ =4.0, *R*_2_ =5.3. The measured results fit reasonably well with the derived Equation S5.


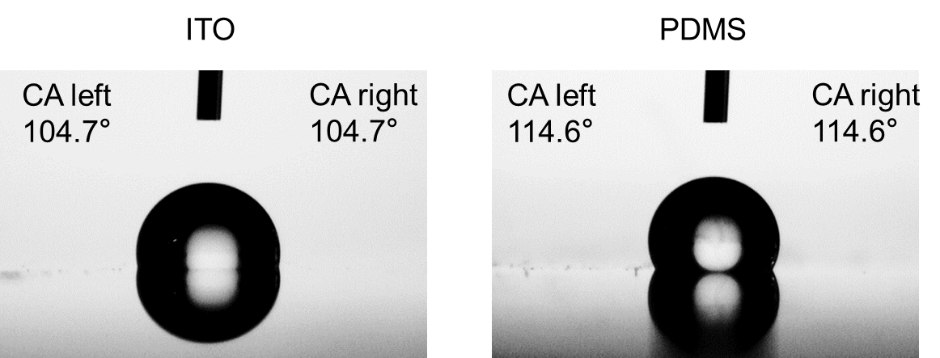


Fig. S10. Photographs of the contact-angle tests. Results including ITO, and PDMS.


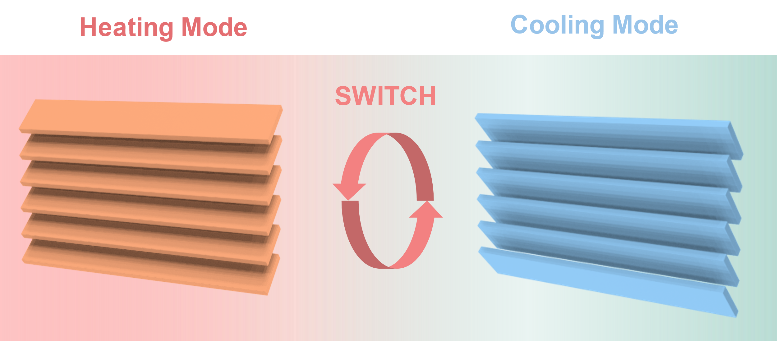


Fig. S11. Equivalent model of kirigami structure as Venetian blinds.


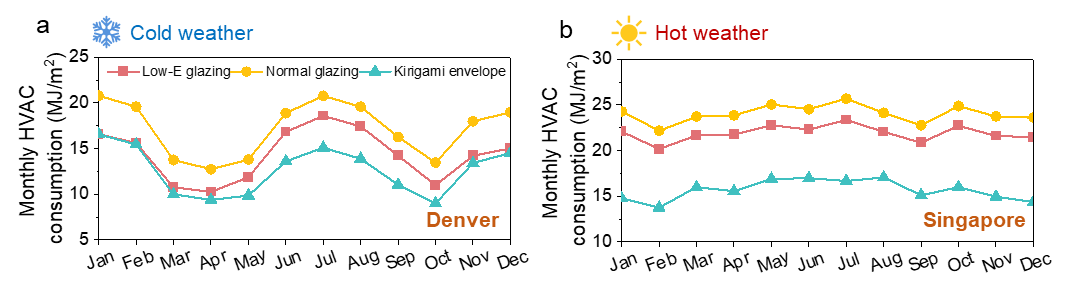


Fig. S12. Monthly HVAC energy consumption of the building based on three different windows in Denver and Singapore.

Table S1. The design of axial layer spacing 𝑦 value

| $L_{C}$ (cm) | $x$ (cm) | $y$ (cm) | $R_{1}=L_{C}/x$ | $R_{2}=L_{C}/y$ |
| --- | --- | --- | --- | --- |
| 14 | 6 | 3 | 2.3 | 4.7 |
| 14 | 6 | 4 | 2.3 | 3.5 |
| 14 | 6 | 5 | 2.3 | 2.8 |

Table S2. Transverse cut length $L_{C}$and transverse cut pitch $x$ value design

| $L_{C}$ (cm) | $x$ (cm) | $y$ (cm) | $R_{1}=L_{C}/x$ | $R_{2}=L_{C}/y$ |
| --- | --- | --- | --- | --- |
| 12 | 8 | 3 | 1.5 | 4.0 |
| 13 | 7 | 3 | 1.9 | 4.3 |
| 14 | 6 | 3 | 2.3 | 4.7 |
| 15 | 5 | 3 | 3.0 | 5.0 |
| 16 | 4 | 3 | 4.0 | 5.3 |

Table S3. Main simulation parameters of equivalent Venetian blinds in simulation

| Project | value | Project | value |
| --- | --- | --- | --- |
| Blind Orientation | Horizontal | Front Side Solar Reflectance | 0.24/0.26 |
| Blind Width (m) | 0.03 | Back Side Solar Reflectance | 0.26/0.24 |
| Blind Separation (m) | 0.024 | Visible Transmittance | 0.66 |
| Thickness (m) | 0.00056 | Front Side Visible Reflectance | 0.13/0.12 |
| Angle (deg) | 45/135 | Back Side Visible Reflectance | 0.12/0.13 |
| Conductivity (W/m·K) | 0.2 | Front Side Infrared Emissivity | 0.95/0.06 |
| Solar Transmittance | 0.5 | Back Side Infrared Emissivity | 0.06/0.95 |
| Blind to Glass Distance (m) | 0.015 |  |  |

Table S4. Representative cities classified by Köppen-Geiger climate [1]

| City | Climate Type | Location |
| --- | --- | --- |
| Singapore | Tropical rainforest | 103.98 E, 1.37N |
| Rio de Janeiro | Tropical savanna | 43.25 W, 22.83 S |
| San Juan | Tropical monsoon | 66 W, 18.42 N |
| Cairo | Tropical and Subtropical Desert | 31.4 E, 30.13 N |
| Denver | Semi-arid | 105.65 W, 39.83 N |
| Arequipa | Cold desert | 71.55 W, 16.32 S |
| Los Angeles | Hot-summer Mediterranean | 118.4 W, 33.93 N |
| Hong Kong | Humid subtropical | 114.17 E, 22.32 N |
| London | Humid temperate oceanic | 0.18 W, 51.15 N |
| Beijing | Hot Summer Continental | 116.47 E, 39.8 N |
| Helena | Warm Summer Continental | 111.97 W, 46.6 N |

**
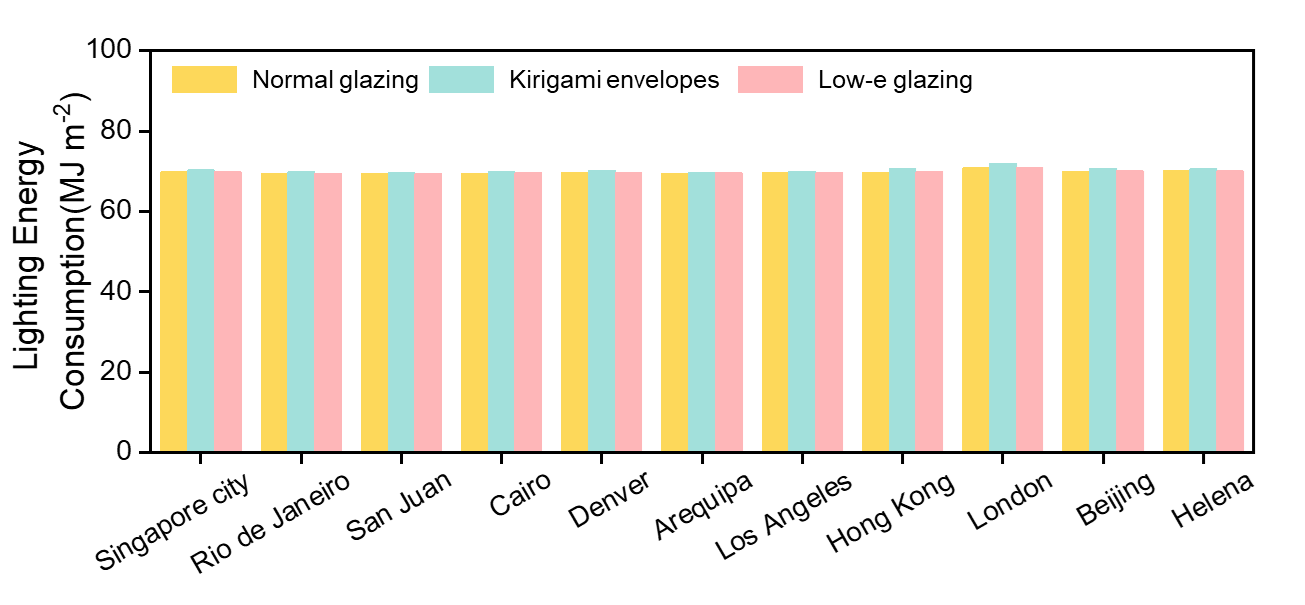
**

Fig. S13. Lighting energy consumption for normal glazing, low-E glazing, and Kirigami envelopes in 11 representative cities, respectively.

*
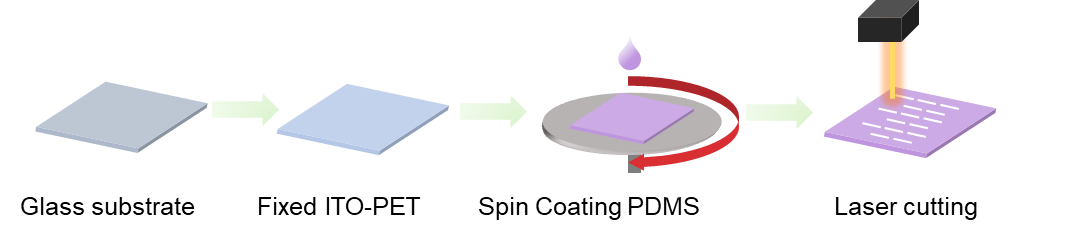
*

Fig. S14. The processing method of kirigami envelopes


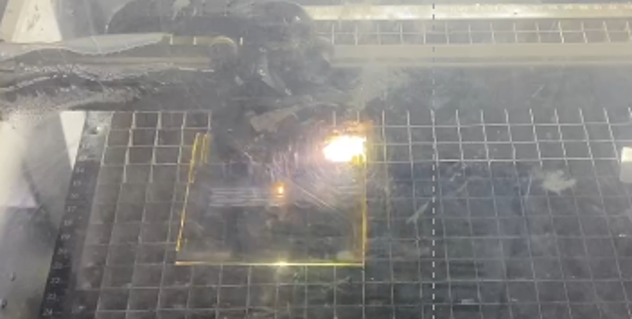


Fig. S15. Kirigami structure laser etching process.

References:

[1] Beck HE, Zimmermann NE, McVicar TR, Vergopolan N, Berg A, Wood EF. Present and future Koppen-Geiger climate classification maps at 1-km resolution. Sci Data. 2018;5:Article 180214.
